# Beneficial effects of the novel first-in-class compound DX243 on ischemic outcomes following *in vitro* and *in vivo* models of stroke

**DOI:** 10.3389/fstro.2026.1802085

**Published:** 2026-06-19

**Authors:** Quentin Marlier, Alexia Boreux, Arnaud Rives, Dario Mosca, Helene Michaux, Louisa Schmitz, Pierre Attali, Stéphane Silvente, Philippe Lefebvre, Brigitte Malgrange, Nicolas Caron

**Affiliations:** 1 Dendrogenix, Liège, Belgium; 2 Laboratory of Developmental Neurobiology, GIGA Institute, University of Liège, Liège, Belgium

**Keywords:** ischemia, MCAO, pharmacological, stroke, treatment

## Abstract

**Introduction:**

New therapeutic strategies to mitigate the devastating consequences of stroke are urgently needed, as restoration of blood flow is currently the only and limited available treatment.

**Methods:**

In our study, we employed a comprehensive combination of *in vitro* and *in vivo* stroke models to investigate the therapeutic potential of the DX243. Specifically, we used glutamate and oxygen-glucose deprivation/reoxygenation (OGD) treatment models to mimic ischemic conditions *in vitro*. To transpose these data *in vivo*, we used the middle cerebral artery occlusion (MCAO) model to induce an ischemic stroke in male C57BL/6J mice.

**Results:**

Our results demonstrated that DX243 significantly increased ATP production following glutamate treatment and neuronal viability following OGD.Forty-eight hours after 1 h of MCAO, mice that received one subcutaneous injection of DX243 presented a reduced infarct volume and preserved motor coordination on the rotarod, while effects on open field locomotion were not statistically significant.

**Conclusion:**

These results highlight the promising neurorestorative properties of this new molecule and suggest its potential as a therapeutic agent for stroke patients. Further investigations in preclinical and clinical settings are warranted to elucidate its full therapeutic potential.

## Introduction

Stroke is the second leading cause of death and the third leading cause of disability worldwide. Approximately 80% of strokes are attributable to cerebral ischemia resulting from a complete or partial occlusion of a cerebral artery. This occlusion leads to a severe reduction in oxygen and glucose delivery to the brain parenchyma perfused by the affected vessel. The middle cerebral artery (MCA) is the most commonly implicated vessel in ischemic stroke (Nogles and Galuska, 2025). This blockage of blood supply leads to an energy failure and irreversible neuronal death within the infarct core. Surrounding this necrotic tissue, the peri-infarct region receives sufficient nutrients to maintain cellular viability; however, it undergoes a delayed pathological cascade over several days. This cascade involves excitotoxicity, synaptic degeneration, mitochondrial dysfunction, and neuroinflammation, ultimately contributing to progressive neuronal loss and an expansion of the ischemic lesion volume (Norat et al., 2020; Wang et al., 2022; Xing et al., 2012). While the infarct core is largely irreversible, mainly due to extensive necrosis, the peri-infarct region remains a therapeutic target owing to its potential for salvage. Currently, the only approved interventions—reperfusion via recombinant tissue plasminogen activator (rtPA) administration or mechanical thrombectomy—aim to restore cerebral blood flow by removing the occluding thrombus. However, this therapeutic strategy does not reduce the consequent neurodegeneration and applies to a limited subset of patients (Bhatia et al., 2020; Ontario, 2016; Xiong et al., 2022; Health Quality, 2016). It is therefore necessary to develop new therapeutic strategies able to reduce stroke-related brain damage and functional consequences.

Here, we analyzed the effect of DX243, a 5α-hydroxy-6β-[3-(4-aminobutylamino)-propylamino]-cholest-7-en-3β-ol small molecule, which is a stable variant of Dendrogenin B (DDB). DX243 has been shown to induce neurite outgrowth in various cell lines, including PC12 and P19 cells, *in vitro* (de Medina et al., 2009). In a guinea pig model, compared with the absence of DX243 treatment, cochlear implant efficacy in the presence of DX243—as assessed by the electrical responsiveness of the auditory nervous system—was associated with functional, but not anatomical, preservation of spiral ganglion neurons. This suggests that DX243 primarily affects neuronal outgrowth (Fransson et al., 2015). More recently, in a rat model of acoustic-trauma–induced cochlear synaptopathy, systemic administration of DX243 (0.05 and 0.1 mg/kg) produced a positive preservative effect on auditory nerve fibers that are particularly vulnerable to aging and acoustic trauma (Fink et al., 2025). Unpublished data have shown that half-life of DX243 in mice is >1 day. DX243 is currently undergoing Phase I/IIa clinical evaluation (clinical trial registration number: 2023-505778-14-00; protocol number: DX243-101).

Based on these promising pharmacological data, we hypothesized that DX243 could also improve neurological outcomes in models of cerebral ischemia and therefore assessed a first proof of efficacy in both *in vitro* and *in vivo* stroke models.

In this study, we first demonstrated that DX243 enhances neuronal survival *in vitro* using the oxygen-glucose deprivation (OGD) model, a well-established paradigm for mimicking ischemic insult (Holloway and Gavins, 2016). Given the persistent uncertainties regarding the mechanisms of cell death following OGD (D'Aes et al., 2023), we employed an alternative *in vitro* model of glutamate-induced neurotoxicity. In this system, we showed that DX243 restores ATP levels in glutamate-exposed neuronal cells, a widely used assay to evaluate compound efficacy in ischemic conditions. To assess the *in vivo* efficacy of DX243, we employed a transient middle cerebral artery occlusion (MCAO) model in male C57BL/6 mice. A single subcutaneous injection of DX243 (1 mg/kg) administered immediately after reperfusion significantly reduced infarct volume. Moreover, DX243 also improved functional outcomes, as evidenced by enhanced motor 48 h post-stroke. Together, these data support the therapeutic potential of DX243 for preserving neuronal integrity and function in ischemic conditions, such as after an acute stroke.

## Materials and methods

### Primary neuronal cell culture

E15.5 pregnant female mice were humanely euthanized, and embryos were removed. Cerebral cortices were then microdissected on ice in PBS-glucose (0.45% glucose) and then transfered into an enzymatic solution containing 0.25% trypsin (T4799-5G, Sigma, St. Louis, MO, USA) and 0.01% DNase (DN25-100MG, Sigma, St. Louis, MO, USA) in EBSS (E2888, Sigma, St. Louis, MO, USA) for 20 min at 37 °C (500 μl/2 cortices). After removing the enzymatic solution, FBS (F7525, Sigma, St. Louis, MO, USA) was added to the cortices for 5 min. Then, the cortices were mechanically dissociated into reduced-serum Opti-MEM (ThermoFisher, Waltham, MA, USA). The resulting cell suspension was filtered through a 70 μm filter. Cell concentration was determined by trypan blue, and cells were plated at a density of 300,000 cells/mL in multi-well plates coated with 40 μg/mL poly-D-lysine (A-003-E, Sigma, St. Louis, MO, USA) and 6 μg/mL laminin (L2020, Sigma, St. Louis, MO, USA). Three hours after plating, half of the culture medium was replaced by fresh Neurobasal medium supplemented with 2% B-27 (17504044, Gibco, Waltham, MA, USA), 2 mM L-glutamine (BE17-605E, Lonza, Basel, Switzerland), and 1% penicillin-streptomycin (DE17-602E, Lonza, Basel, Switzerland). The resulting primary cortical neurons were maintained in culture for 5 days (DIV5) in a humidified incubator at 37 °C with 5% CO_2_ and 95% air until the experiments.

### Oxygen-glucose deprivation/ reoxygenation

Ischemia-like conditions were induced in primary cortical neurons at day *in vitro* 5 (DIV5) by replacing the Neurobasal medium with glucose-free DMEM (11966-025, Gibco, Waltham, MA, USA), followed by incubation in a hypoxic chamber (1% O_2_, 5% CO_2_,94% N_2_). Control neurons were kept in neurobasal medium supplemented with 2% B-27, 2 mM L-glutamine and 1% penicillin-streptomycin under normoxic conditions (5% CO_2_ and 95% air) for the same duration. After 4h of OGD, reoxygenation was initiated by replacing the glucose-free DMEM medium with neurobasal medium supplemented with 2% B-27, 2 mM L-glutamine and 1% penicillin-streptomycin and incubating the cells under normoxic conditions (5% CO_2_, 95% air) for up to 24 h. During this reoxygenation phase, cells were treated with DX243 at 1nM or 100 nM, or left untreated.

### Immunocytochemistry cell viability

24 h after OGD, cell survival was analyzed by immunostaining. Cultures were fixed by replacing the culture medium with 4% paraformaldehyde for 10 min. After two washes with PBS for 5 min, coverslips were then incubated overnight in PBS containing Tween-20 (0,1%), Triton (0,1%) with Tuj1 mouse (1:500, Cell Signaling #4466, Danvers, MA, USA) and rabbit Cleaved-caspase 3 (1:500, Cell Signaling #9661T, Danvers, MA, USA) primary antibody diluted in 5% goat serum (Cell Signaling #5425, Danvers, MA, USA). Coverslips were then washed 3 times in PBS before incubation for 1 h at room temperature in PBS containing Tween (0,1%), Triton (0,1%) with anti-mouse (1:2000, Cell Signaling #4408, Danvers, MA, USA) and anti-rabbit (1:2000, Cell Signaling #4413, Danvers, MA, USA) secondary antibodies. Finally, coverslips were washed 3 times in PBS and mounted with the antifade prolonged gold DAPI (Cell signaling, Danvers, MA, USA). Images were collected using a confocal microscope (Nikon A1 system) and quantitative analysis was performed by counting 40x random fields until reaching a minimum of 100 DAPI^+^ cells. Cell viability was assessed as the normalization to normoxic condition (Nx-NT) of the ratio between living and dead DAPI cells, where discrimination between living and dead cells was considered according to their DAPI morphology (Nguyen et al., 2007). Living cells exhibit bigger, less bright, and uniform morphology compared to brighter (condensed), smaller, and irregular dead cells morphology. To verify that no apoptotic cells could be considered as DAPI living cells, we co-stained neuronal cultures with cleaved-caspase-3. Tuj1 labeling has been used as an illustration of the neuronal culture shape with no quantification.

### Trypan blue cell viability

24 h following reperfusion, neuronal survival was also evaluated by trypan blue analysis. Medium from every well of a 24-well plate was collected into separate 5 mL Falcon tubes. After medium removal, 250 μL of 0.25% trypsin was added to each well for 10 min at 37 °C. Then, 250 μL of FBS was added for 5 min before adding 500 μL of Neurobasal medium. One mL of each cell suspension was then centrifuged for 5 min at 400 g. Cells were resuspended in 100 μL of Neurobasal medium, and the number of viable cells was evaluated by diluting 10 μL of the cell suspension into 90 μL of trypan blue (T8154, Merck), followed by counting on the TC20 Automated Cell Counter (Bio-Rad, Hercules, CA, USA) and normalized on the Nx-NT condition.

### SH-SY5Y cell culture

Human neuroblastoma SH-SY5Y (C0005004, Clinisciences) were cultured in DMEM/F-12 medium (11330-032, Gibco, Waltham, MA, USA) supplemented with 10% FBS and 1% penicillin/streptomycin (Gibco, Waltham, MA, USA) in a humidified incubator supplied with 95% air and 5% CO_2_ at 37 °C. Cells were passaged once a week with trypsin 0.25% (CA TRY-3B, Westburg, Leusden, Netherlands) and medium was changed three times a week.

### Glutamate treatment

SH-SY5Y cells were seeded in 96-well plates with 100 μL at 175 000 cells/mL for 72 h. The cells were divided in 5 subgroups of 100 μL treatment: (1) treated with culture medium only (untreated or NT); (2) treated only with sodium L-glutamate monohydrate (G0188, TCI, Tokyo, Japan) at 80 mM; (3) cotreated with 80 mM of glutamate and 1 nM of DX243; (4) cotreated with 80 mM of glutamate and 1 mM of creatine (B25009.22, ThermoFisher, Waltham, MA, USA) and (5) cotreated with 80 mM of glutamate and 5 μM of D-AP5 (HY-100714A, medchem express, Monmouth Junction, NJ, USA). Creatine and D-AP5 were included as reference compounds with known anti-excitotoxic properties, serving as experimental positive controls (Genius et al., 2012; Minnella et al., 2018). D-AP5, a selective NMDA receptor antagonist, was used to counteract glutamate-induced excitotoxicity. After 24 h of incubation, the medium was replaced with glutamate-free medium, and the cells were subsequently exposed for an additional 24 h to DX243 (1 nM), creatine (1 mM), or D-AP5 (5 μM).

### Luminescent cell viability assay

Intracellular ATP levels were quantified using the CellTiter-Glo^®^ 2.0 Cell Viability Assay (G9241, Promega, Madison, WI, USA) with minor modifications. In brief, 50 μL of the CellTiter-Glo^®^ 2.0 reagent was added to 100 μL of cell suspension. The mixture was incubated for 2 min under agitation in the dark, followed by a 10-min incubation at room temperature. Luminescence was recorded with a 1-s integration time using a TriStar^2^S LB 942 plate reader (Berthold). Relative ATP levels were calculated as the ratio of the relative light units (RLU) of treated samples to those of untreated controls, expressed as a percentage.

### Animals

The reporting of this study complies with the ARRIVE guidelines (ARRIVE 2.0) (Percie du Sert et al., 2020):

8 weeks C57BL/6J male mice (Janvier labs) were group-housed (up to 5 per cage) in the animal facility at the University of Liège under standard conditions, with food and water *ad libitum*, and maintained on a 12-h light/dark cycle.

All animals were cared in accordance with the guidelines of the Belgian Ministry of Agriculture, in agreement with the EC laboratory animal care and use regulation Directive 2010/63/EU, 22 September 2010 and the relevant Belgian implementing legislation (e.g.„ the Royal Decree of 29 May 2013 on the protection of animals used for scientific purposes. The Animal Care Ethics Committee of the University of Liège approved all experiments (Ref 18-2060).

Adult male mice were randomly assigned to four experimental groups using a computer-generated randomization list with block sizes chosen to ensure balanced representation of each group on each surgery day (Table 1; mean weight 22.47 g, *n* = 123). The four groups consisted of MCAO mice with or without DX243 treatment and SHAM mice with or without DX243 treatment, allowing evaluation of both the effects of MCAO and the impact of the treatment. Inclusion and exclusion criteria were predefined as follows: mice that died before the behavioral assessment at 48 h post-surgery, mice that did not exhibit a reduction in cerebral blood flow as measured by laser Doppler flowmetry (LDF), and mice that did not present an infarct area on TTC staining were excluded from the study. These criteria ensured that animals in which a stroke was not successfully induced were not included in the analyses. Because no consensus exists regarding the exact threshold of LDF reduction required after MCA occlusion (Morris et al., 2016), LDF was used primarily to confirm correct filament placement during surgery, with the filament advanced until a clear decrease in blood flow (>40%) was observed. The great majority of animals experienced a reduction in CBF of >70% (21/24) when a small proportion of animals with MCAO drops falling between 60 and 70% (2/24) and 1 animal with a value at 42%.

**Table 1:** Number of included/excluded mice.

Groups	Total (*n*)	Average CBF drop (%)	Average Surgery duration all mice (min)	Weight (g)			Exclusion				Average Surgery duration included mice (min)	Final (*n*)
				Average (±SD)	Min	Max	Mortality		No LDF drop	No infarct (TTC-based)		
							BR	AR				
SHAM CTL	14	n.a	17.3	22.56 (±0.74)	21.2	23.8	2	n.a	n.a	n.a	17.5	12
SHAM DX243	12	n.a	17.9	22.52 (±0.66)	21.2	23.8	0	n.a	n.a	n.a	17.9	12
MCAO CTL	47	84.7	21.5	22.61 (±0.46)	21.95	23.25	9	8	2	16	20.8	12
MCAO DX243	50	85.6	21.0	22.38 (±0.77)	20.8	23.45	8	14	0	16	20.8	12

BR, before reperfusion; AR, after reperfusion.

Two mice were excluded because no reduction in LDF was detected, most likely due to incomplete filament insertion.

An a priori sample size calculation was performed where the primary endpoint for power calculation was infarct volume (percentage of ipsilateral hemisphere) between MCAO-treated and vehicule mice. A power calculation was performed to be able to detect a 30% reduction in infarct volume with a variance of 33% (80% power and an alpha-error of 5%) whereas behavioral outcomes were exploratory, using the same groups that were powered primarily for infarct volume. Anticipating substantial attrition due to the MCAO model (mortality and failed occlusions), we planned an initial group size larger than 12 to account for anticipated exclusions.

All investigators involved in MCAO surgeries, injections, or behavioral assessments were blinded to the treatment groups. All animals were allowed to acclimatation 24 h before rotarod training, 48 h before rotarod baseline and 6 days before surgeries.

### Middle cerebral artery occlusion

Mice received a subcutaneous injection of buprenorphine (0.05 mg/kg) 20 minutes before anesthesia was induced with 2% isoflurane. Animals were then placed in a chemical hood on a heating pad to maintain body temperature (without precise temperature control). All surgeries were performed under an operating stereomicroscope using aseptic technique (sterile instruments and aseptic preparation of the neck region).

Regional cerebral blood flow was monitored using a laser-Doppler flowmeter (Model 1639; Moor Instruments Ltd., Axminster, UK). A small incision was made in the scalp to expose the right side of the skull. A drop of Loctite surgical glue (Loctite + accelerator; Moor Instruments, UK) was applied at the cortical region irrigated by the middle cerebral artery (approximately 1–2 mm posterior and 4–5 mm lateral to Bregma), and single-fiber Doppler probe tips were secured to the bone using Loctite surgical glue and accelerator (Loctite + accelerator; Moor Instruments, UK).

Mice were then turned to the supine position, and a midline cervical incision was made to expose the right common carotid artery (CCA) adjacent to the trachea. The proximal and distal CCA, as well as the external carotid artery, were ligated using 5-0 silk sutures. A small arteriotomy was made between the ligatures on the CCA, and a 6-0 coated monofilament (Doccol, Sharon, MA, USA) was introduced and advanced through the internal carotid artery to the origin of the MCA (10–11 mm from the carotid bifurcation) until a reduction in cerebral blood flow was detected via laser-Doppler.

After closure of the neck and scalp incisions, mice were allowed to recover for 1 h in a warmed cage. They were then re-anesthetized, placed on a heating pad, and the neck incision was reopened to expose the CCA. The monofilament was withdrawn to allow reperfusion (without LDF monitoring). Mice were allowed to recover for an additional 5 h in a warmed cage, with access to jelly food and moistened pellets. Buprenorphine (0.05 mg/kg) was administered again between 4 and 6 h and at 24 h after surgery.

The number of animals included in the analysis or excluded is reported in Table 1. Sham mice underwent an identical procedure except that the coated monofilament was not inserted into the MCA.

### DX243 treatment

For *in vitro* experiments, DX243 was used as a water-soluble salt (e.g., trichloride form), prepared at 10 mM in adjusted pH aqueous buffer. The solution was prepared using vortex and/or sonication, if applicable, and visually inspected to ensure absence of precipitation. DX243 was used at final concentrations of 1, 10, or 100 nM in the culture medium. In the OGD model, DX243 was added at DIV5 immediately after the hypoxic period and maintained throughout the 24-h reperfusion phase. In the glutamate excitotoxicity assay, DX243 was applied for 48 h: initially during the 24-h glutamate exposure period, and subsequently for 24 h following glutamate removal.

For *in vivo* experiments, DX243 was dissolved in 0.9% NaCl at 1 mg/mL with the same quality control for clarity and absence of precipitation. Mice received a single subcutaneous injection of DX243 (0.2 mg/mL; 1 mg/kg) immediately after filament removal (i.e., 1-h post-MCAO). DX243 was freshly prepared weekly, stored at 4 °C, and protected from light throughout the experimental period.

### Infarct volume measurement

Mice were euthanized 48 h post-reperfusion by cervical dislocation followed by decapitation to ensure death prior to sample collection. The brain was removed from the skull, and 2-mm coronal sections were made into a brain matrix (RBMS-200C, WPI) from the end of the olfactory bulb to the beginning of the cerebellum to obtain 3 to 4 sections. Sections were placed in PBS/5% D-glucose before being transferred into a 1% TTC solution (T8877, Sigma, St. Louis, MO, USA) for 20 min under gentle agitation. The stained sections were captured with a digital camera.

To correct for ischemia-induced edema, infarct volumes were quantified using ImageJ (Supplementary Figure S1) according to Reglogi's method described by Nouraee et al. (2019).

Contralateral and ipsilateral-non-infarcted brain volumes were first determined by averaging the anterior and posterior areas of each slice and multiplying by the slice thickness (2 mm). The volumes of all slices were then summed to obtain the total contralateral and ipsilateral-non-infarcted brain volumes. Infarct volume was calculated in the same manner: the mean infarct area from the anterior and posterior views was multiplied by the slice thickness (2 mm), and these values were summed across slices. The resulting infarct volume was corrected for edema by applying the scaling factor [contralateral volume divided by total ipsilateral volume (infarcted + non-infarcted)]. The infarct-to-brain ratio was then calculated by dividing the corrected infarct volume by twice the total contralateral volume. When tissue loss or incomplete slicing occurred, a virtual boundary was drawn using the contralateral hemisphere as a reference.

### Behavioral assessment

All behavioral assessments were performed 48 h after MCAO surgery, following a 30 min habituation period in a testing room devoid of natural light. Illumination was provided by an overhead white neon lamp located above the apparatus, with light intensity measured at approximately 75 lux using the *Luxmeter (Coolexp)* application on a Samsung Galaxy A36.

### Open field

Spontaneous locomotor activity was evaluated using the open field (OF) test. This test measures total distance traveled, mean velocity, and percentage of time spent moving, with time spent in the center zone used as an exploratory indicator of anxiety-like behavior. The OF apparatus consisted of a beige ABS-plastic arena (40 cm × 40 cm × 30 cm; 3201 mouse open field, Maze Engineers Inc., Boston, MA, USA). Each mouse was placed in the arena and allowed to explore freely for 3 min.

During the session, the center of mass of each animal was tracked using EthoVision XT software (Noldus), which automatically calculated locomotor parameters (total distance, velocity, and percentage of time spent moving). The apparatus was cleaned with 10% ethanol and dried between trials to eliminate olfactory cues.

### Rotarod

Motor coordination and balance were assessed using an accelerating rotarod apparatus (Maze Engineers). The testing session lasted a total of 4 min and consisted of four consecutive 1-min phases with increasing speeds and acceleration settings as follows: Minute 1: Speed 5 rpm / Acceleration 10. Minute 2: Speed 10 rpm / Acceleration 20. Minute 3: Speed 20 rpm / Acceleration 30. Minute 4: Speed 30 rpm / Acceleration 40. Mice were pre-trained on the rotarod with three trials per day for two consecutive days (4 and 3 days prior to MCAO surgery). Forty-eight hours after surgery, latency to fall was recorded as the primary outcome measure of motor coordination.

### Western blot

Following TTC staining quantification, ischemic hemispheres from Sham and MCAO mice were immersed in ice-cold lysis buffer (RIPA) and sonicated to homogenize the brain samples. Homogenates were incubated for 15 min and centrifuged at 12,000 x G for 15 min (4 °C). Protein concentration was measured using the BCA assay (23225, ThermoFisher, Waltham, MA, USA), following the manufacturer's protocol. Proteins were diluted to 25 μg/μL in water and Laemmli (4X) (1610737, Bio-Rad, Hercules, CA, USA) in the presence of 1 μL of Total protein reagents (A44449, Invitrogen, Waltham, MA, USA). Proteins were heated at 95 °C for 2 min, and 20 μL of the solution was separated on a 4–12% gel (4561083, Bio-Rad, Hercules, CA, USA) and transferred to a PVDF immunoblotting membrane (4561083, Bio-Rad, Hercules, CA, USA). The membranes were blocked with 3% BSA in Tris-buffered saline with Tween-20 (TBS-T) for 1 h, then incubated overnight at 4 °C with anti-NeuN (1:500, Cell Signaling #94403, Danvers, MA, USA) and anti-PSD95 (1:500, Cell Signaling #3450, Danvers, MA, USA) primary antibodies in 3% BSA/TBS-T. Membranes were then incubated for 45 min at RT with mouse secondary antibodies conjugated to horse radish peroxidase (1:2000, Cell Signaling #7076, Danvers, MA, USA). Pierce ECL WB substrate (32209, ThermoFisher, Waltham, MA, USA) was used to detect immunoreactive bands.

Protein expression levels were measured using ImageJ software by analyzing the intensity of each band on Western blot images. Signal intensities for each protein of interest were first normalized to the total protein content of the corresponding lane (e.g., total protein stain). For each experimental condition (SHAM, MCAO, and MCAO + treatment), the normalized values were then expressed as a percentage relative to the wild-type control (WT), which served as an internal reference. Identical WT protein samples were loaded on each gel to enable inter-gel normalization across independent experiments.

### Statistical analysis

All statistical analyses were conducted using GraphPad Prism 8.0 (GraphPad Prism Software^®^). All data were tested for normality using the Shapiro–Wilk test. Data with a Gaussian distribution were analyzed with parametric tests and shown as mean ± SD. Data that did not exhibit a Gaussian distribution were analyzed with a nonparametric test (Kruskal-Wallis) and reported as median with interquartile range. Results were considered statistically significant at *p* ≤ 0.05. For comparisons involving more than two groups, one-way ANOVA was performed followed by post hoc pairwise comparisons with the Bonferroni correction for the percentage of living cells relative to Nx-NT (IF), behavioral outcomes, and western blot quantification. Two-way ANOVA was used for the percentage of cell survival relative to Nx-NT (trypan blue) and percentage luminescence relative to CTL (CellTiter-Glo^®^ 2.0). The unpaired *t*-test was employed for direct comparison between two groups. For analysis and comparison of Kaplan–Meier survival curves, the log-rank (Mantel–Cox) test was used.

## Results

### DX243 increases neuronal survival in primary cortical neurons subjected to oxygen-deprivation

**Figure 1:**
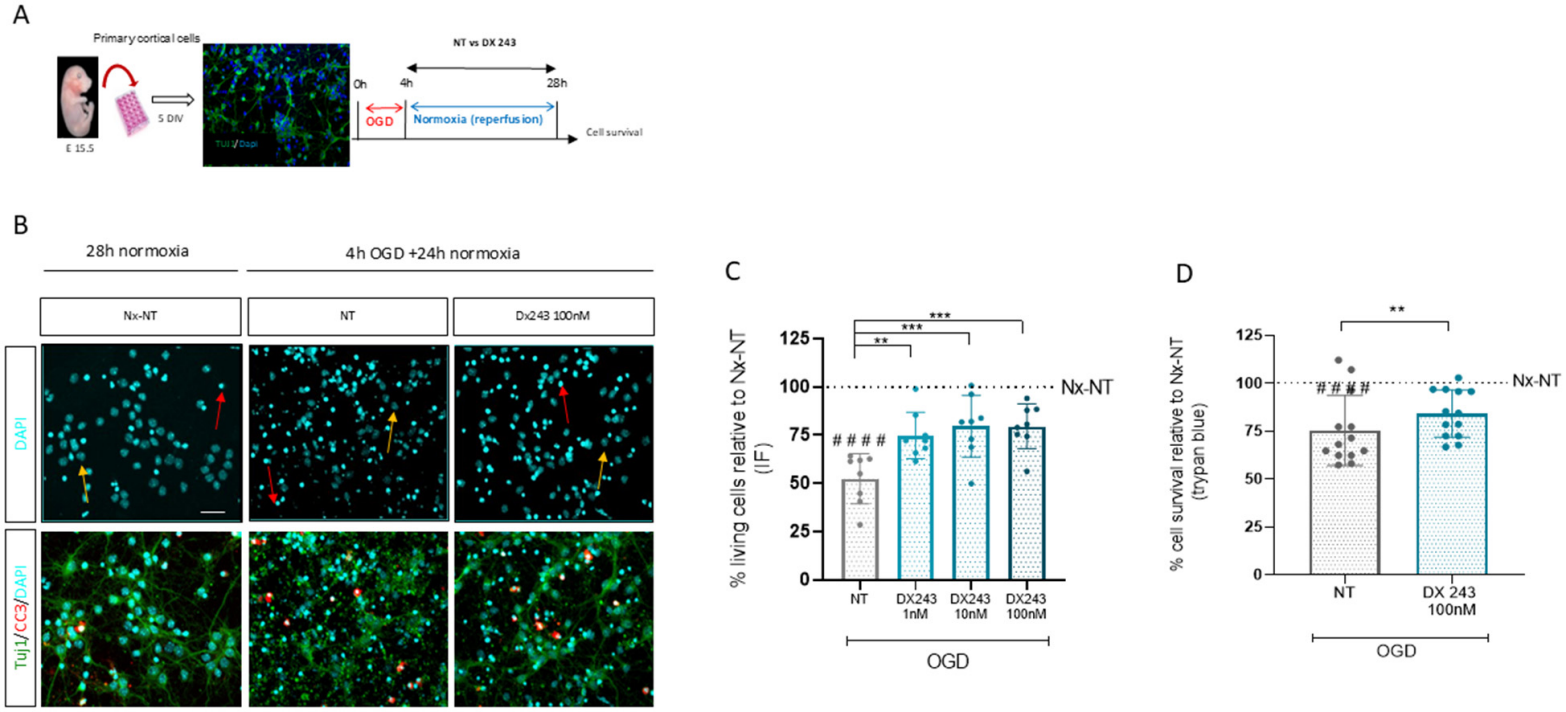
DX243 increases neuronal survival in the OGD model. **(A)** Scheme of the experimental protocol. **(B)** Representative confocal microscopy images showing cell survival increased (yellow arrows—Cell morphology with DAPI staining) in OGD DX243-treated primary cortical cells and showing apoptosis (CC3) and neuronal health (Tuj1 staining), scale bar=30 μm **(C)** Quantification of viable neuronal cells (DAPI living cells/All DAPI cells) relative to normoxia condition (Nx-NT); *n* = 8 (8 cultures from 8 different pregnant mice). **(D)** Quantification of living cells by trypan blue relative to normoxia condition (Nx-NT); *n* = 17 (17 wells from 5 different pregnant mice). Data are presented as mean ± SD; **p < 0.01 and ***p < 0.001 = treated condition vs. NT and ^*####*^ < 0.0001=NT vs. Nx-NT condition.

To evaluate the neuroprotective potential of DX243 under ischemic-like conditions, we assessed its effects on neuronal survival following oxygen-glucose deprivation (OGD) in primary cortical neurons. Following 5 days of differentiation, primary cultures of cortical neurons (composed of 95% of neurons, and 5% of astrocytes) were subjected to OGD, followed by a reperfusion phase in the presence or absence of DX243 at 1, 10, or 100 nM. Quantification of viable neuronal cells was then assessed using the ratio of DAPI living and dead cells determined through their morphology. Merged pictures with Tuj1 (B-tubulin III), Cleaved-caspase-3 (CC3), and DAPI illustrate the absence of living DAPI cells co-stained with CC3, whereas Tuj1 labeling illustrates the health of the neuronal culture (Figure 1A).

Following OGD exposure, confocal imaging (Figure 1B) revealed improved neuronal morphology (DAPI) and reduced apoptosis (CC3) in DX243-treated cultures compared to untreated OGD controls (NT), along with preserved neuronal identity (Tuj1 staining). Quantification of DAPI-positive viable neurons (Figure 1C) revealed a significant decrease in cell viability to 52.3% in NT relative to normoxic condition (Nx-NT). DX243 treatment at 1 nM significantly increased cell viability from 52.3% to 74.8%, while 10 nM and 100 nM of DX243 both increased cell viability from 52.3% to 79.5%. These results were corroborated by trypan blue exclusion assay (Figure 1D), which showed a significant viability reduction to 73.1% following OGD as compared to Nx-NT conditions. DX243 treatment at 100 nM significantly increased cell viability from 73.1% to 82.5%. These findings demonstrate that DX243 confers significant neuroprotection against OGD-induced injury by enhancing neuronal survival and preserving cellular integrity in a dose-dependent manner.

### DX243 improves ATP levels following glutamate treatment in SH-SY5Y neuronal cell line

**Figure 2:**
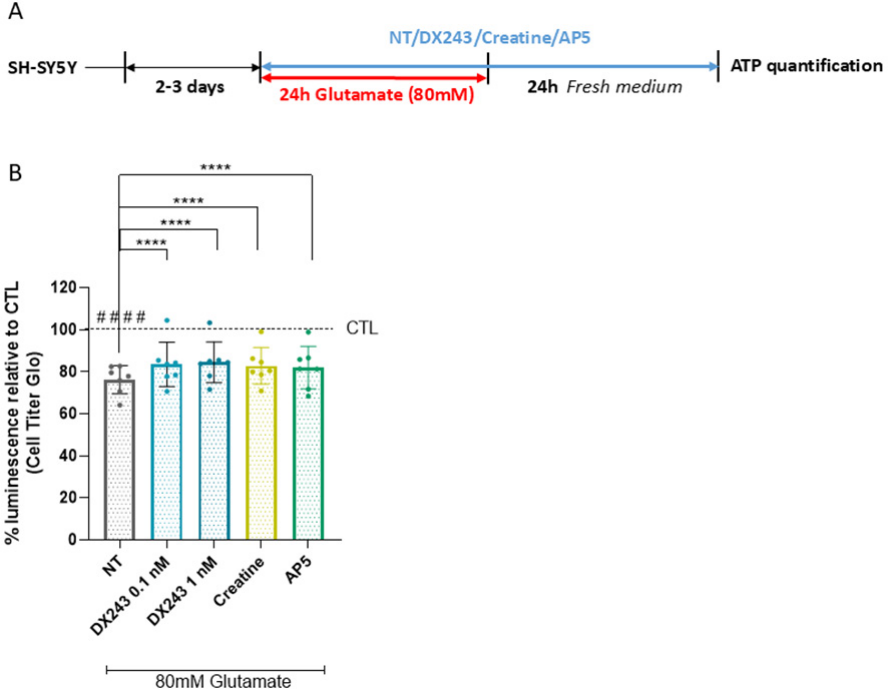
DX243, Creatine and AP5 increases ATP levels following glutamate treatment. **(A)** Scheme of the experimental protocol. **(B)** ATP levels quantification measured by CellTiter-Glo^®^ 2.0 Cell Viability Assay (relative luminescence) in SH-SY5Y cells non-treated (CTL) or exposed to 80 mM of glutamate (24 h) in the presence of DX243 (1 nM), or both positive controls Creatine (1 mM) and AP5 (5 μM). Data are represented as mean ± SD with each experiment consisted of multiple wells (6 wells) per condition, and the *n* = 7 shown corresponds to 7 independent experiments (different cell passage), each normalized to its own (from the same independent experiment) untreated control = 100%; ****p < 0.001 = treated condition vs. NT and ^*####*^ < 0.0001 = NT vs. Nx-NT condition.

To confirm the beneficial effects of DX243 on neuronal cells under ischemic-like culture conditions, SH-SY5Y neuronal cells were exposed to 80 mM glutamate for 24 h (Palanivel et al., 2022) in the presence or absence of DX243. 80 mM has been selected based on glutamate dose-response experiments (Supplementary Figure S2). Following glutamate exposure, a post-treatment phase was conducted by replacing the medium with fresh culture medium, again with or without DX243 (Figure 2A). Cellular ATP content, used as an indicator of cell viability, was quantified using the CellTiter-Glo luminescence assay.

Upon glutamate treatment, ATP levels in SH-SY5Y cells significantly decreased to 76.2% relative to untreated control (NT) conditions. Treatment with Creatine (1 mM) and D-AP5 (5 μM), both used as positive controls due to their protective effects against glutamate toxicity (Genius et al., 2012; Minnella et al., 2018), significantly increased ATP levels from respectively 76.2% to 82.8% (Creatine) and 82% (D-AP5), thereby validating the excitotoxicity model. DX243 at 0.1 nM and 1 nM significantly increased ATP levels from respectively 76.2% to 83.5% and 84.5%, demonstrating a neuroprotective effect comparable to that of the positive controls (Figure 2B).

### DX243 reduces ischemic lesion volume

**Figure 3:**
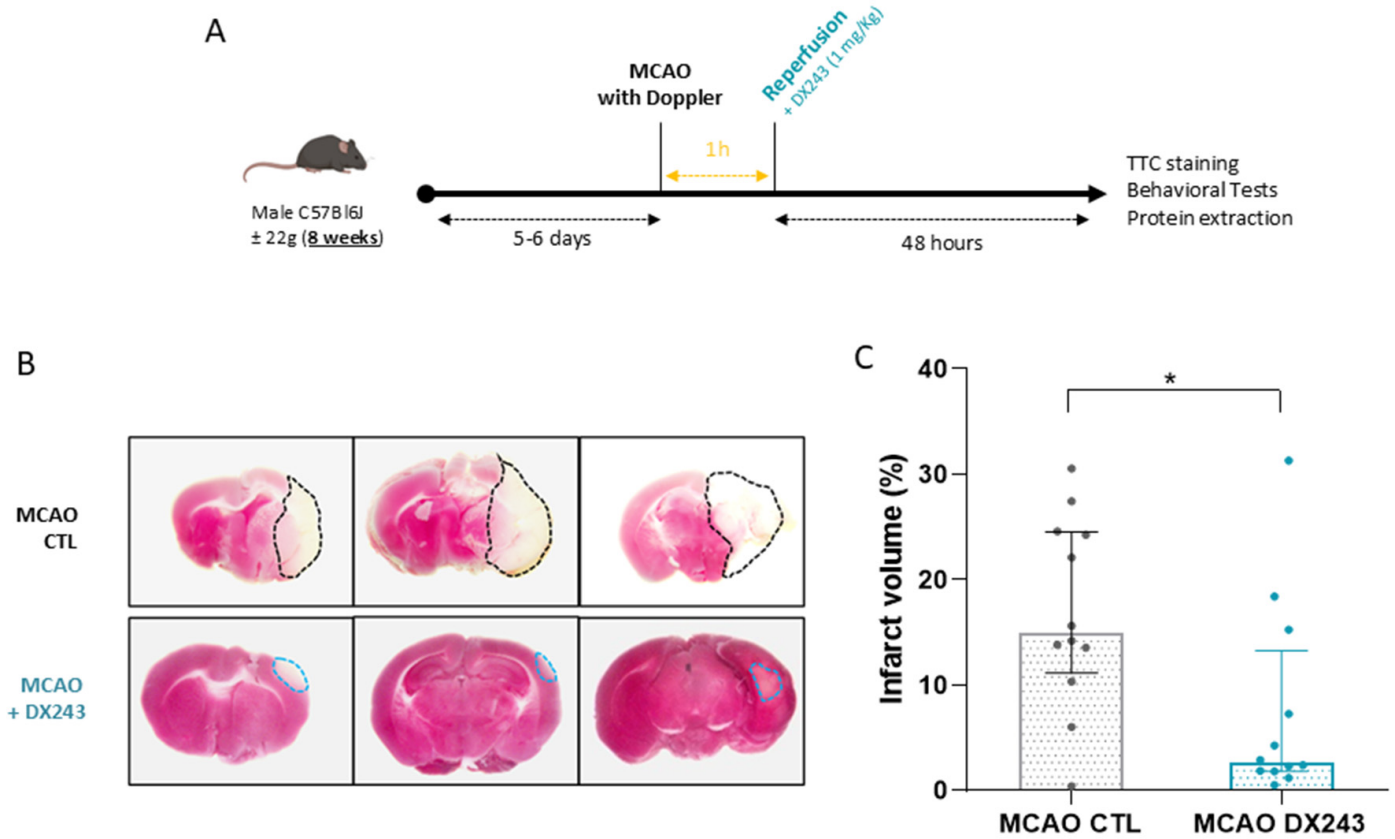
DX243 reduces ischemic lesion volume **(A)** Scheme of the experimental protocol. **(B)** Representative pictures of TTC-stained brain slices from Ctrl or DX243-treated mice 48 h following MCAO. **(C)** Quantification of brain infarct volume in MCAO CTL and DX243-treated mice showing a significant decrease (81.9%) of brain infarct volume in DX243-treated mice. Data are represented as median ± interquartile range with *p < 0.05 and *n* = 12 mice/group.

To assess the neuroprotective potential of DX243 *in vivo*, we first quantified infarct volume in a murine model of focal cerebral ischemia induced by MCAO (Figure 3A). Infarct volume was assessed on brain sections using TTC staining 48 h post-occlusion. DX243-treated mice exhibited a 81.9% relative reduction in infarct volume compared with the untreated group, with median values of 14.9% (MCAO CTL) and 2.7% (MCAO + DX243) (Figures 3B,C).

### DX243 preserves forced motor coordination after MCAO

**Figure 4:**
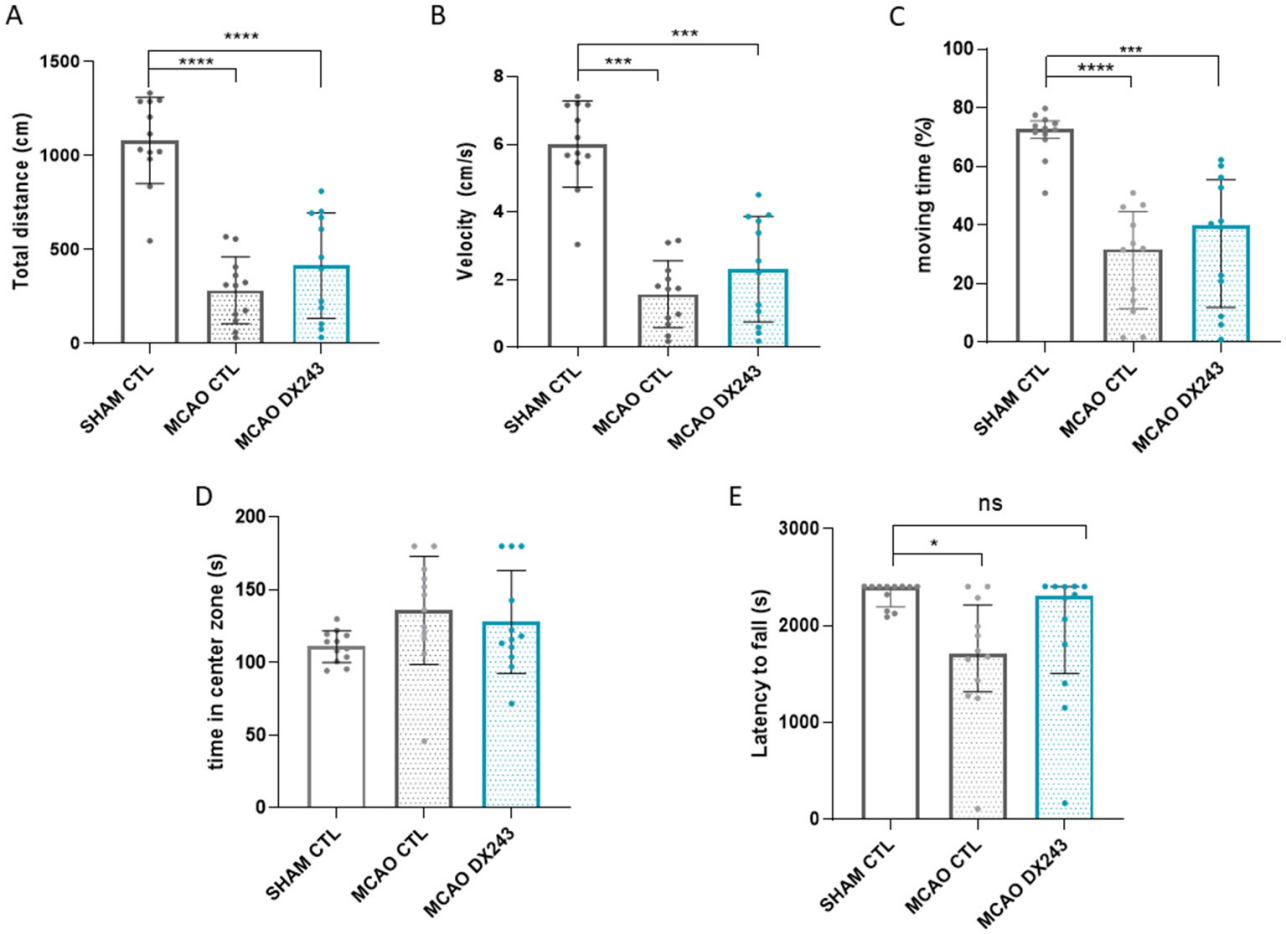
DX243 improves functional outcomes following 1 h MCAO. **(A)** Histograms showing the total distance traveled (3 min) in open field by sham or MCAO mice treated or not with DX243 at 1 mg/kg. MCAO mice presented a significant decrease in total distance, but DX243-treated mice showed no statistical increase of spontaneous locomotion activity measured in the open field. Data are represented as mean ± SD and *n* = 12 mice/group. **(B,C)** Histograms showing the velocity and the % of moving time performed in open field (3 min) by sham or MCAO mice treated or not with DX243 1 mg/kg. MCAO mice showed a significant decrease in both velocity and % of moving time, but DX243-treated mice showed no statistical increase of velocity and % moving time, respectively. Data are represented as mean ± SD **(B)** or as median ± interquartile range **(C)** with ***p < 0.001 and ****p < 0.0001. **(D)** Histograms showing the time spent in the central zone of the open field by sham or MCAO mice treated or not with DX243 at 1 mg/kg. No differences were observed in the time spent in the central zone. Data are represented as mean ± SD and *n* = 12 mice/group. **(E)** Histograms showing the latency to fall measured in sham or MCAO mice treated or not with DX243 at 1 mg/kg. Compared to SHAM animals, DX243-treated mice showed no statistical difference in motor coordination measured by the latency to fall quantification in Rotarod. Data are represented as median ± interquartile range with *p < 0.05 and *n* = 12 mice/group.

We next assessed the functional consequences of DX243 treatment on mice's motor performance following MCAO. Spontaneous locomotor activity was evaluated using the open field test. As shown in (Figure 4), MCAO induced a marked impairment in motor behavior, evidenced by a significant reduction in total distance traveled (from 1080.7 cm to 282.7 cm, corresponding to a 73.8% decrease) (Figure 4A), accompanied by decreased movement velocity (from 6 cm/s to 1.6 cm/s, corresponding to a 73.3% decrease) and a lower percentage of time spent moving (from 72.8% to 31.8%) (Figures 4B, C). Although the total distance increased from 282 cm in MCAO-untreated mice to 415 cm in DX243-treated mice, mean velocity improved from 1.6 cm/s to 2.3 cm/s and movement time increased from 31.8% to 39.9%, none of these differences reached statistical significance. The time spent in the center zone of the open field did not differ between SHAM and MCAO groups (Figure 4D), suggesting no significant anxiety-related behavior. Motor coordination was further assessed using the rotarod test at 48 h post-MCAO. CTL mice exhibited a significant pronounced decline in performance compared to SHAM, as measured by the latency to fall from the rotarod, while DX243-treated mice showed no statistical difference compared to SHAM mice. Figure 4E, indicating partial preservation of motor coordination. Notably, DX243 had no effect on spontaneous locomotion or motor performance in SHAM-operated animals (Supplementary Figure S3). These results indicate that DX243 partially preserves motor function after cerebral ischemia, supporting its therapeutic potential to improve post-stroke outcomes.

### DX243 reduces neuronal loss and increases synaptic levels after MCAO

**Figure 5:**
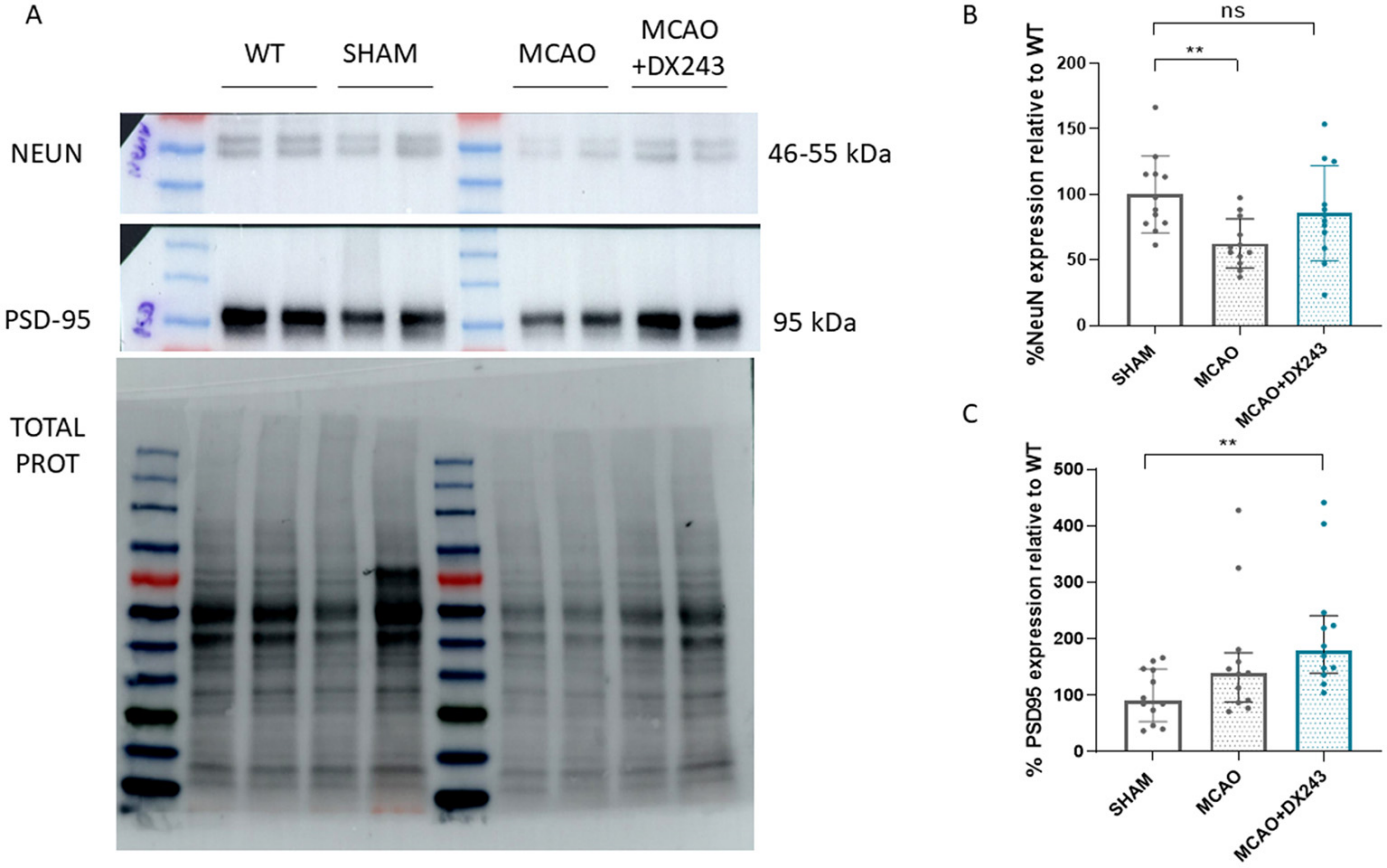
Western-blot analysis of NeuN and PSD95 in the ischemic brain after MCAO. **(A)** Representative western-blot of NeuN, PSD-95 and **(B, C)** quantification of both NeuN and PSD-95 expression in SHAM, MCAO CTL and MCAO DX243-treated mice. Two protein samples from unoperated mice (referred as WT; same age, sex, and strain) were loaded in every western-blot. Six WB were then performed with these 2 WT samples (always same samples for each western-blot) + 2 different SHAM, 2 different MCAO CTL and 2 different MCAO DX243 to obtain 12 expression values per group (*n* = 12mice/group) normalized on the mean of the 2 WT samples. Protein level expression normalized on total protein level. Data are represented as mean ± SD **(B)** or as median ± interquartile range **(C)** with **p < 0.01.

We investigated the effects of DX243 treatment on neuronal survival and synaptic integrity following cerebral ischemia. To this end, western blot analyses were performed (Figure 5A) on the ischemic hemispheres of three groups of mice: Sham-operated controls (SHAM), untreated MCAO mice (MCAO), and MCAO mice treated with DX243 (MCAO + DX243). To quantify neuronal and synaptic alterations across these conditions, we measured the expression of NeuN and PSD-95. NeuN is a nuclear protein selectively expressed in mature neurons and serves as a marker of neuronal survival, while PSD-95 is a postsynaptic scaffolding protein indicative of synaptic integrity. In MCAO mice, NeuN expression was significantly reduced by 37.4% compared to SHAM animals, confirming ischemia-induced neuronal loss. DX243 treatment increased NeuN levels from 62.6% (MCAO CTL) to 85.7% (MCAO + DX243) (Figure 5B), with no statistical difference between DX243-treated mice and SHAM mice suggesting a neuroprotective effect. Similarly, PSD-95 expression was significantly increased from 89.7% to 178% in MCAO + DX243 mice compared to SHAM mice (Figure 5C), indicating a beneficial effect of DX243 on synaptic structures.

## Discussion

In this study, we demonstrate that DX243, a novel first-in-class clinical compound, exerts a robust neuroprotective effect across complementary *in vitro* and *in vivo* models of ischemic injury. DX243 enhanced neuronal survival under oxygen deprivation in primary cortical neurons, restored ATP production following glutamate exposure in SH-SY5Y cells, markedly reduced infarct volume after MCAO, and preserved motor coordination. At the cellular level, DX243 limited neuronal loss and preserved synaptic integrity in the ischemic hemisphere. Together, these findings identify DX243 as a promising bioenergetic modulator that mitigates the multifaceted consequences of cerebral ischemia.

Our *in vitro* results provide mechanistic insight into how DX243 may confer neuroprotection. The increased survival of primary cortical neurons subjected to oxygen deprivation suggests enhanced cellular tolerance to severe metabolic stress. Additionally, the restoration of ATP levels following glutamate exposure indicates that DX243 efficiently counteracts excitotoxic energy depletion. As these two paradigms model distinct aspects of ischemic pathophysiology, the consistent benefit of DX243 across both supports a mechanism centered on maintaining neuronal bioenergetics.

Although glutamate concentrations used in excitotoxicity assays vary considerably across studies, largely depending on the cell type, culture conditions, and experimental objectives (Kritis et al., 2015), the glutamate concentration used in our model (80 mM) is higher than those most commonly reported in the literature for SH-SY5Y (Palanivel et al., 2022). However, the concentration used likely reflects a limitation inherent to the SH-SY5Y excitotoxicity model rather than an attempt to exaggerate the insult. Our preliminary dose–response experiments demonstrated that lower concentrations, including 40 mM, failed to induce a robust and reproducible ATP depletion, resulting in high variability and insufficient sensitivity to detect post-treatment neuroprotective effects. Thus, while the use of 80 mM glutamate may reduce direct comparability with some published studies, it was necessary to achieve a stable, quantifiable, and reproducible excitotoxic challenge compatible with pharmacological evaluation in our experimental conditions (Supplementary Figure S2).

The *in vivo* findings further highlight the functional relevance of this bioenergetic support as DX243 significantly reduced ischemic lesion volume. This structural protection translated into preservation of motor coordination, indicating preservation of neural circuits essential for motor behavior. The alignment between histological protection and behavioral performance strengthens the evidence that DX243 confers meaningful therapeutic potential. The divergence between the beneficial effects observed on the rotarod and the absence of improvement in spontaneous locomotion likely reflects both the severity of the ischemic injury and the inherent differences between these behavioral assays. The rotarod represents a forced motor task that requires sustained engagement of balance, coordination, and motor drive. Because animals are compelled to perform the task, it can partially overcome the functional impact of stroke severity, thereby reducing the apparent deficit and increasing the likelihood of detecting a treatment effect. In contrast, the open field test assesses self-initiated locomotion, a behavior strongly influenced by post-stroke apathy, reduced motivation, or general sickness behavior. As such, spontaneous exploratory activity is more vulnerable to the suppressive effects of ischemic injury, making treatment-related improvements more difficult to detect in this paradigm.

Histopathological analyses also show that DX243 preserves key cellular components affected by ischemia. The reduction in neuronal loss within the ischemic hemisphere reflects protection of vulnerable neuronal populations, while increased synaptic marker levels suggest maintenance of synaptic architecture. Because synaptic degeneration contributes significantly to long-term functional decline after stroke, the preservation of synaptic integrity likely supports the behavioral improvements observed in DX243-treated animals.

Overall, these convergent findings suggest that DX243 reduces ischemic damage by maintaining neuronal energy metabolism during and after ischemic insult, without establishing a direct causal mechanism.

This mechanism is different from traditional neuroprotective strategies that target excitotoxicity or inflammation and may overcome some limitations that have prevented the translation of previous neuroprotective agents (Chia et al., 2023; Yang et al., 2019) Coupled with its favorable pharmacokinetic profile and proven safety from hearing-loss programs, DX243 presents a promising candidate for therapeutic development in acute ischemic stroke.

Some limitations should be considered. In line with the exploratory nature of this work, the present study should be considered a first proof-of-concept evaluation aimed at determining whether DX243 could provide neuroprotection in ischemia-relevant *in vitro* and *in vivo* models. This stroke-focused investigation is complementary to, but distinct from, the preclinical development program that supported entry of DX243 into clinical evaluation for presbycusis. Given that the rationale for testing DX243 in stroke emerged from pharmacological effects initially observed in hearing-loss models, this study was intentionally designed as an initial, limited assessment of its therapeutic potential prior to undertaking a full STAIR-compliant preclinical program. DX243 needs now to be evaluated in another preclinical study to suit with STAIR rules (Bix et al., 2018; Bosetti et al., 2017; Crossley et al., 2008; Lyden et al., 2023; Sena et al., 2010). Indeed, long-term functional outcomes and combination approaches with reperfusion therapies should be investigated in future work. Moreover, in addition to morphometric studies such as MRI scans, other behavioral tests will be necessary to evaluate the effects of DX243 on sensitive, motor, and cognitive functions, such as mazes or beam walking tests. It is also strongly recommended to show the DX243 effect in animals with co-morbidities typical of stroke patients, such as diabetes and hypertension, or in aged animals.

The attrition rate in this study was higher than usually described (Lee et al., 2014; Smith et al., 2015), which we now recognize as a limitation that may affect the robustness and generalizability of the findings. However, to assess whether mortality may have influenced the interpretation of our results, we generated a Kaplan–Meier survival curve comparing MCAO control animals and DX243-treated animals. No significant difference in survival was observed between the two groups (*p* = 0.3496), indicating that mortality is unlikely to have biased the functional or histological outcomes reported in this study regarding DX243-treatment effect (Supplementary Figure S4).

Considering surgery time as a potential variable influencing the data, we performed a statistical analysis of the surgery duration for all included mice (Supplementary Figure S5) which showed no difference between the MCAO CTL group (20.8 min) and the MCAO DX243 group (20.8 min), indicating that the effects of DX243 are unlikely to be biased by differences in procedure duration. However, a significant difference was observed between the SHAM CTL group (17.5 min) and the MCAO CTL group, suggesting that surgical duration may have influenced the MCAO CTL group relative to SHAM CTL. To avoid this potential source of bias, SHAM procedures should ideally be intentionally prolonged to match MCAO surgical duration.

While an acclimation period of 6 days before surgery is generally considered adequate, a limitation of this study is that rotarod training began 24 h after animal arrival, representing a relatively short habituation period. However, because the rotarod is a forced motor coordination task rather than a spontaneous behavioral assay, it is less sensitive to mild transport-related stress. This likely minimizes the impact of the shortened acclimation on the interpretation of motor performance outcomes.

The exact molecular mechanism underlying ATP restoration remains to be determined, and comprehensive pharmacokinetic and pharmacodynamic studies in the context of stroke are required.

Importantly, DX243 demonstrated efficacy when administered as a treatment since it was injected at the end of the occlusion, at the time of reperfusion. Given that currently only an estimated 10–20% of patients receive thrombectomy, due to clinical contraindications or delayed hospital arrival, the therapeutic effect observed after stroke induction is particularly promising.

In conclusion, DX243 preserves neuronal survival, maintains synaptic integrity, and improves functional recovery following cerebral ischemia. Its demonstrated safety, favorable biodistribution, and complete subcutaneous bioavailability further support the translational relevance of DX243 as a novel therapeutic approach in acute ischemic stroke.

## Data Availability

The original contributions presented in the study are included in the article/Supplementary material, further inquiries can be directed to the corresponding author.
