# Supplementary material for: Beneficial effects of the novel first-in-class compound DX243 on ischemic outcomes following in vitro and in vivo models of stroke
Source: Front Stroke. 2026 Jun 19;5:1802085. doi: 10.3389/fstro.2026.1802085 (PMC13330481; doi:10.3389/fstro.2026.1802085)
Supplement: Supplementary file 1 [file Supplementary_file_1.docx]

Supplementary Material

**
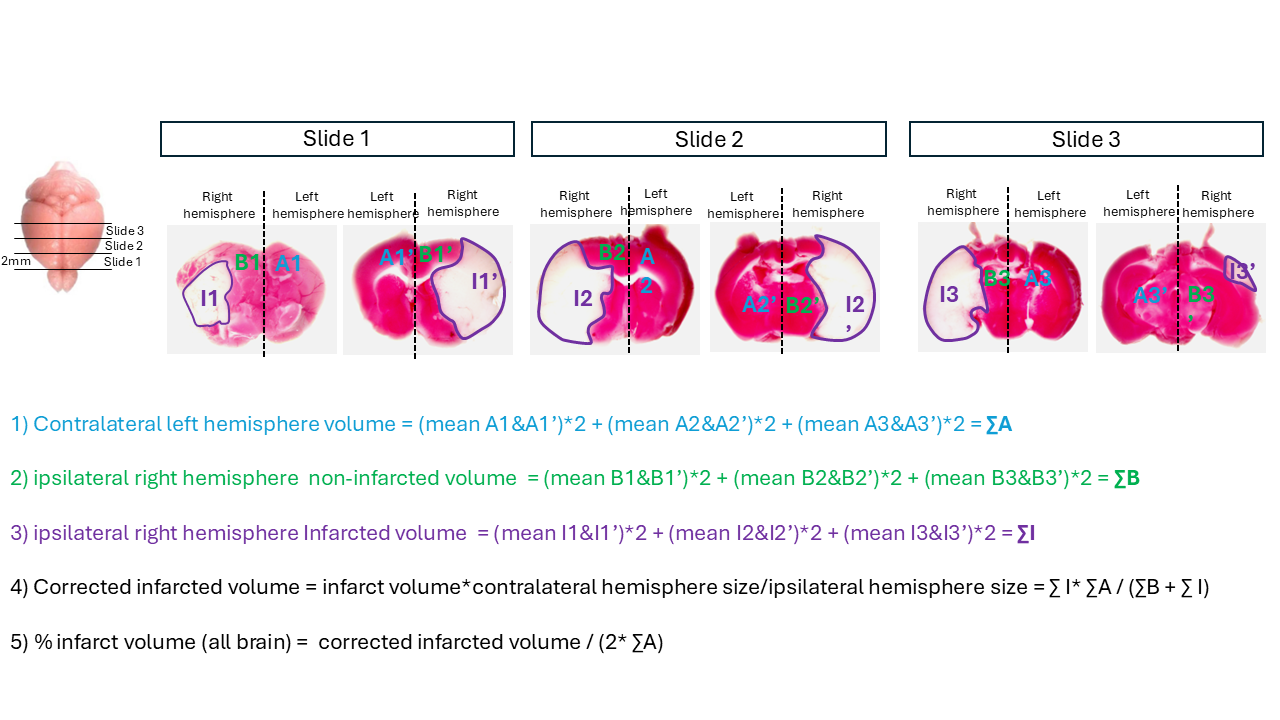
**

**Supplementary Figure 1**. Infarct volume calculation - Explanation of how the volume of the ischemic lesion was quantified while accounting for lesion-induced edema.

**Supplementary Figure 2. Optimization of glutamate concentration to induce excitotoxicity in SH‑SY5Y cells.** Relative luminescence units (RLU) were measured after exposure to glutamate (24h treatment + 24h wash out) at the indicated concentrations (40–200 mM) and normalized to the non‑treated condition (NT), demonstrating that 80 mM of glutamate induces a stable, quantifiable, and reproducible excitotoxic challenge while preserving a measurable therapeutic window for the evaluation of DX243. Data are represented as mean ± SD; n =3-5 independent experiments; One-way ANOVA; **p<0.01;****p<0.0001 compared to NT condition.


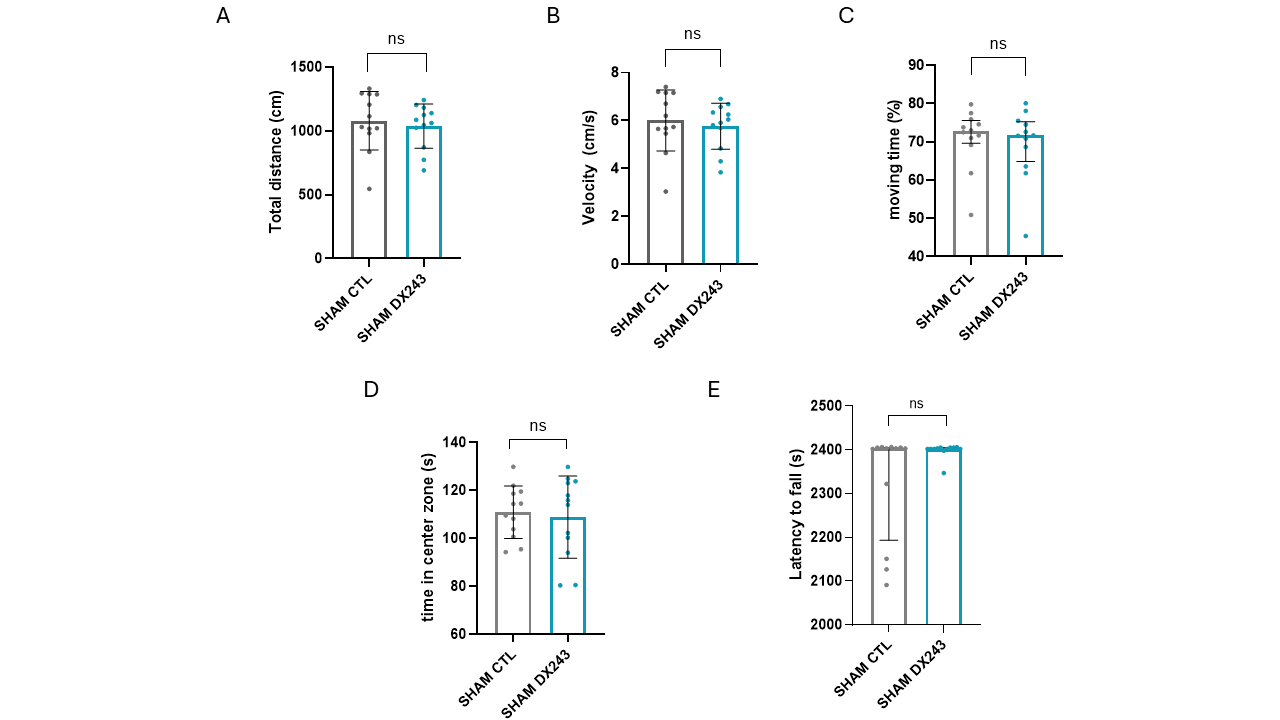


**Supplementary Figure 3**. DX243 does not influence motor behavior in Sham mice. **(A-B)** Histograms showing the total distance traveled and the velocity in the open field (3min) performed by sham mice treated or not with DX243 at 1 mg/kg. DX243-treated mice showed no modified spontaneous locomotion activity or velocity. Data are represented as mean ± SD and n =12 mice/group. **(C)** Histogram showing the % of moving time performed in the open field (3 min) by sham mice treated or not with DX243 1mg/kg. DX243-treated mice showed no modified % of moving time. Data are represented as median ± interquartile range; n =12 mice/group. **(D)** Histogram showing the time spent in the central zone of the open field by sham mice treated or not with DX243 at 1 mg/kg. No differences were observed in the time spent in central zone. Data are represented as mean ± SD and n =12 mice/group. **(E)** Rotarod histograms showing the latency to fall in sham-operated mice treated or not with DX243 at 1 mg/kg. DX243 that treatment did not modify motor coordination, as indicated by unchanged latency to fall. Data are represented as median ± interquartile range; n =12 mice/group.

**Supplementary Figure 4**. Kaplan–Meier survival curves show the proportion of surviving animals over the 48‑h period following middle cerebral artery occlusion (MCAO). Survival curves were compared using the log‑rank (Mantel–Cox) test; p=0.3496 indicates no statistically significant differences between groups.

**Supplementary Figure 5.** Histogram showing surgical time between the MCAO CTL, MCAO DX243, SHAM CTL and SHAM DX243. Data are represented as mean ± SD and n =12 mice/group, showing statistical difference between SHAM CTL and MCAO CTL; One-way ANOVA; *p<0,05.
